# Behaviour of a Premixed Flame Subjected to Acoustic Oscillations

**DOI:** 10.1371/journal.pone.0081659

**Published:** 2013-12-20

**Authors:** Shafiq R. Qureshi, Waqar A. Khan, Robert Prosser

**Affiliations:** 1 Engineering Sciences Department, Pakistan Navy Engineering College, National University of Sciences and Technology, Karachi, Pakistan; 2 School of MACE, University of Manchester, Manchester, United Kingdom; German Cancer Research Center, Germany

## Abstract

In this paper, a one dimensional premixed laminar methane flame is subjected to acoustic oscillations and studied. The purpose of this analysis is to investigate the effects of acoustic perturbations on the reaction rates of different species, with a view to their respective contribution to thermoacoustic instabilities. Acoustically transparent non reflecting boundary conditions are employed. The flame response has been studied with acoustic waves of different frequencies and amplitudes. The integral values of the reaction rates, the burning velocities and the heat release of the acoustically perturbed flame are compared with the unperturbed case. We found that the flame's sensitivity to acoustic perturbations is greatest when the wavelength is comparable to the flame thickness. Even in this case, the perturbations are stable with time. We conclude that acoustic fields acting on the chemistry do not contribute significantly to the emergence of large amplitude pressure oscillations.

## Introduction

Thermoacoustic instabilities result from the uncontrolled amplification of acoustic waves during combustion. These instabilities are more apparent in combustion systems operating on a lean premixed air fuel ratio, and several mechanisms for the instability have been identified (i.e. [1], [2], [3], [4], [5], [6], [7], [8]). Although combustion systems are normally designed for steady state conditions, some regions of the operating envelope may be prone to the growth of instabilities arising from small initial disturbances. Although these disturbances consume only a very small part of the available energy in the chamber, large pressure oscillations may follow, leading to structural vibrations and—in extreme cases—- “equipment failure” [9].

The noise arising from unsteady combustion is commonly expressed in terms of a thermoacoustic Efficiency (TAE), defined as
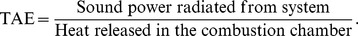
Typical values of the TAE for turbulent flames are 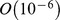 and for laminar flame are 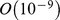 [3], [10], [11]. Instabilities occur for thermoacoustic efficiencies of 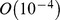 [10]; for every order of magnitude change in the TAE, the sound pressure level (SPL) changes by about 10 dB [11].

A small acoustic wave propagating through the flame may be altered either in amplitude or frequency and this may effect the combustion dynamics. The direct influence of acoustic wave propagation on reaction rates to our knowledge has not been discussed in the literature separately. However, the effect of a wave propagating through a non-equilibrium background has been discussed by numerous authors. Einstein (cited in [12]) and Clarke & McChesney [13] suggest that wave attenuation may occur in dissociating mixtures when the wave itself drives the non-equilibrium component of the flow. Elaine et al. [12] describe how frequency dispersion emerges when a sound wave alters its shape while propagating through a non-equilibrium background. Furthermore, they suggest that acoustic wave amplification is expected only if the non-equilibrium flow already exists in the background, or is caused by an external source and not by the propagating wave itself. Clarke [14] has shown that the non-equilibrium background flow can indeed amplify the acoustic wave . Experimental work by Toong et al. [15] has shown evidence of both the amplification and the suppression of sound waves when they interact with a flame, although these observations are based upon a diffusion flame. Similar conclusions have also been drawn by Melvin [16], Srinivasan & Vincenti [17], and Bauer & Bass [18].

The focus of this paper is, therefore, to study the response of a premixed laminar methane flame to small acoustic disturbances and to identify which—if any—acoustic modes induce positive feedback in the pressure oscillations. The novelty of the work comes from the relative complexity of the reaction mechanism employed (18 species and 68 individual reaction steps), and the configuration studied (Low Mach number flow, with fully non-reflective inlet and outlet boundary conditions).

Section provides a review of flame-acoustic interaction and reaction rate chemistry. The governing equations, discretization schemes and boundary condition treatment for reacting flows are given in section 0.3, along with a brief description of the code used. Results of the simulations are presented in section 2, and conclusions are presented in section 0.6.3.

## Acoustic Waves and Reaction Rates

A generalized inhomogeneous wave equation can be derived to describe the relationship between the pressure and heat release fluctuations in an acoustically active field such as a combustion chamber. In the combustion chamber, the source of heat release is solely due to the chemical reactions between oxidizer and fuel. Any acoustic perturbation in the combustion chamber will interact with the flame and may modify the flame structure substantially [19]. Sound generation due to heat release has been reviewed by Higging, Sondhauss and Rijke; an account of their work is given in [20]. Numerous authors (i.e. Putnam and Dennis [21], Shimmer and Vortmeijer [22]) have undertaken experimental studies to investigate flame-acoustic interactions. Putnam et al. [21] have also provided a mathematical formulation for the development of these acoustic instabilities .

The generation of acoustic waves in a flame may be due to a natural mode of system, the addition of energy by an external source or by chemical reactions within the system [12]. An order of magnitude analysis of a turbulent reacting mixture shows that heat release fluctuations driven by the species reaction rates 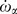 provide the dominant sources [23]. The inhomogeneous acoustic wave equation governing reacting flows involving *N* chemical species can be expressed in the following form [23], [12]:
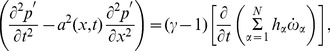
(1)where 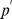 is the pressure fluctuation, 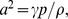 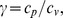 and 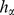 is the species enthalpy, defined as
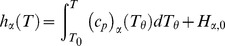
with 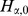 taking the value of the reference state enthalpy. 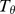 here is an integration variable. The reaction rate for species 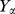 is derived by considering *I* elementary reactions between *N* species;
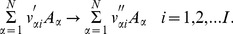
(2)

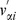
 and 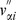 are the stoichiometric coefficients for species 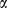 during reaction step 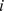, and 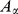 represents the chemical species. 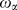 is then given by
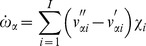
with
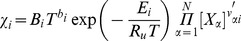
(3)The term 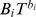 represents the collision frequency and is often known as the frequency factor or pre-exponential factor, *E* is activation energy [24]. The values of 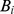, 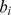 and 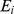 are empirical parameters and are based on the nature of the elementary reactions. The activation energy is the energy required to move the reactants over the energy barrier to begin the reaction [25]. 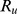 is universal gas constant. 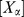 representing the molar concentration of species 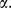 For reversible reactions, 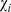 is modified with the addition of an analogous term describing the backwards rate of reaction. This may be specified explicitly as part of the reaction mechanism, or derived via equilibrium considerations.

## Simulation

To study the effect of acoustic waves on flame chemistry, a number of simulations have been carried out using an in-house code. The code is based around a fully compressible solver and was initially developed to study multidimensional reacting flows with arbitrarily complex reaction mechanisms. For the purposes of this work, the problem is specified as one dimensional. Explicit 4th order spatial differencing was employed to calculate the derivatives appearing in the transport equations, while time integration was handled via the low storage 3rd order Runge Kutta scheme proposed by Wray [26]. Prior to this study, the code has been validated against a number of test problems, as recommended by Roache [27], and has been used in a number of other test cases.

### 0.1 The governing equations

The governing equations for a compressible viscous reacting flow can be written in the following form:
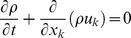


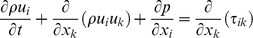


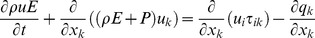


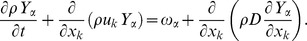
Where tensor indices i,k = 1,2,3. The transport equations are closed via the thermal equation of state, and the stagnation energy relation [28]

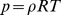


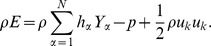
The viscous stress tensor is defined as
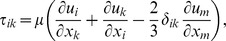
and 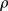, 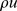, 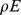, 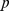, 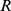 are the density, momentum, total energy, pressure and characteristic gas constant, respectively. The effects of gravity and radiative heat transfer are assumed to be negligible [29], [30]. The heat flux 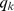 is given by
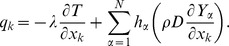
Lewis and Prandtl numbers are considered constant in this study [29], [30]. Therefore the mass diffuivities 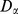 of each species and viscosity are derived via assumption of constant Lewis and Prandtl numbers using following expressions:
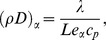


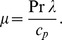
The value for 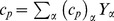 is obtained using the CHEMKIN thermodynamic database for the constituent specific heat capacities 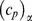 [31], and the thermal conductivity is assumed to be given by
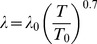
(4)


### 0.2 Boundary conditions

Boundary conditions for flows within a finite domain (i.e. closed ducts) are relatively straightforward to treat. In the case where the flow domain is infinite and unbounded, a truncation of the physical domain is desirable for a numerical solution, but such a truncation requires an artificial boundary. Since the focus of our study is to investigate the behaviour of acoustic waves passing through a flame, and since any reflection from the inlet or outlet boundaries may produce spurious effects, we use non reflecting boundary conditions based upon the method of characteristics.

The method of characteristics describes how systems of hyperbolic equations can be decomposed into sets of wave modes, each with a definite velocity [32]. At each boundary of the computational domain, some waves enter the domain and some waves leave the domain. The outgoing waves are entirely defined by the interior solution. The incoming waves depend on the exterior solution and require a boundary condition. Thompson [32] gives a complete mathematical analysis and describes the incoming and outgoing waves in a primitive variable form for the Euler equations. This approach has been extended by Poinsot and Lele [33] for the application of non-reflecting boundary conditions to the Navier-Stokes Equations. This approach is commonly referred to as the Navier–Stoke Characteristics Boundary Conditions (NSCBC) approach. An application of this method to reacting flows was initially proposed by Baum et al. [28] and later extended by Sutherland and Kennedy [34].

Further refinements to the NSCBC approach have been proposed by Prosser [35], who used a two-scale low Mach number expansion [36] to identify a linearization based around a divergence free state for cold flows. These have been extended to include conducting and reacting flows [37]. For the boundary conditions used in this study, we effectively set
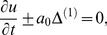
(5)where the sign depends on the boundary under consideration, 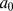 is the sound speed based on the far field base-state and 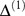 is the *acoustic divergence*, defined as [37]

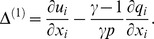

Equation 5 thus specifies an inflow boundary condition which is fixed, modulo the passage of acoustic transients. Details regarding development and implmentation of NSCBC for reacting flows can be seen elsewhere [33], [35], [37], [32]

### 0.3 Discretization schemes, chemistry, and boundary conditions

A one dimensional domain of length 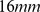 is discretized using 1024 nodes, resulting in a grid spacing of 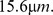 The reaction zone (flame thickness) is approximately 4 mm long. An explicit 4th order finite difference method is used for the spatial discretization of the continuity, momentum, energy and species transport equations [38]

**Figure 1:**
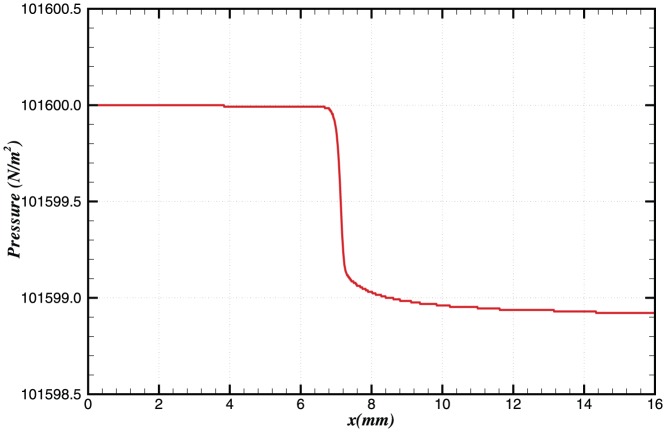
Steady state pressure profile in the domain.

**Figure 2:**
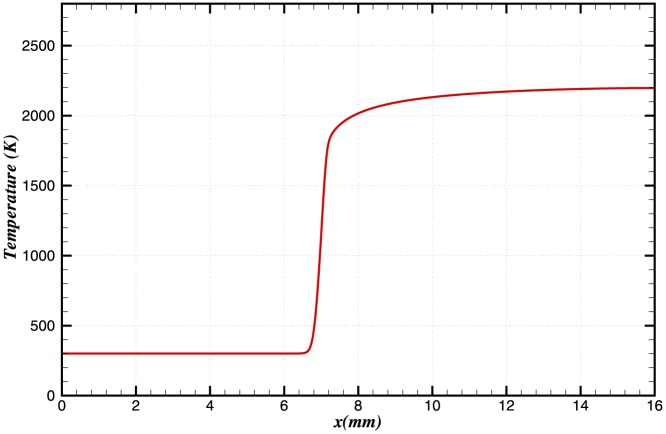
Steady state temperature profile in the domain.

A methane mechanism comprising 68 reaction steps and 18 species is used for the source terms in the species transport equations. The specific heat capacities, enthalpy and entropy are calculated using the polynomial coefficients of the CHEMKIN thermo chemical tables [31]. The simulation is initiated using assumed profiles for key species, and then allowing the calculation to proceed until all of the dependent variables have approached a steady state. By setting the inlet mass flow rate equal to the consumption rate, a stationary flame solution is achieved; this is used as the initial condition for the acoustically active simulation. All simulations are performed assuming an equivalence ration 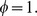 The pressure and temperature profiles of the steady state solution are shown in Figure 1 and Figure 2. The equilibrium flame temperature is approximately 2200 K, and the flame speed is calculated to be 0.32 m/s

The acoustic wave trains directed toward the flame are generated by manipulating the incoming characteristics. For a quiescent field with no significant viscous effects or chemical reactions, it is straightforward to show that the left (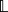) and right 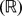 going acoustic amplitudes may be related via
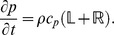
At the left hand boundary, we set
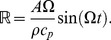
(6)Tthe boundary condition produced by equation 6 produces a wave train of amplitude *A* and frequency 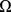 on the inlet plane.

## Results and Discussions

We are interested in the interaction between the acoustic field and the reaction zone. The coupling between the chemistry and the acoustics can manifest itself in one of two ways

- There may exist an amplification/attenuation of the wave as it passes through the flame; such a finding would be consistent with the proposition of Clarke et al. [14], [13]. This will be examined in the next section
- The pressure gradients induced by the incoming wave train may effect reaction rates of different species in the flame structure; this in turn could couple the wave to the reaction rate, and set up a resonance. This will be examined in section 0.6.

From the flame's perspective, low frequency waves induce negligibly small pressure gradients on the length scales associated with the reaction zone. In such cases, it is extremely unlikely that lighter species could be preferentially displaced with reference to the heavier species. Hence, we have selected relatively high frequency ranges, up to the point where the acoustic wavelength is of the same order as the flame itself; typically, this is around 90 kHz. These latter frequencies are beyond those typically encountered in industrial applications; our interest in them here stems from the fundamental physics.

### 0.4 Single wave propagating through a non-equilibrium background

**Figure 3:**
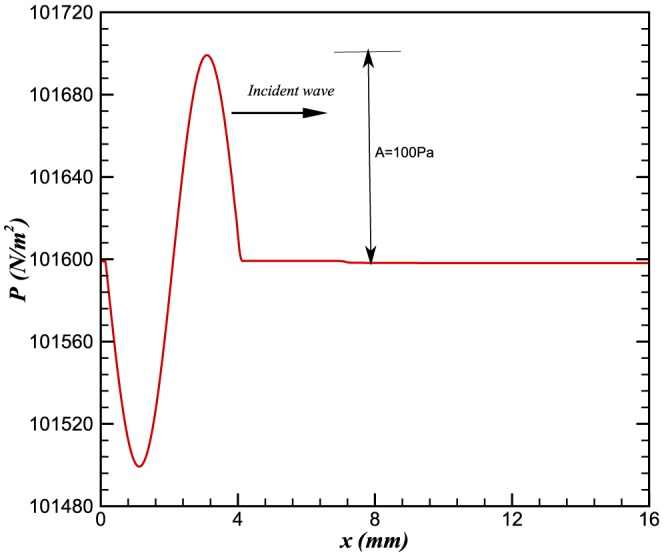
Incident wave of 90 kHz.

The presence of the flame in the domain acts almost as a discontinuity in the flow due to the sudden changes in density, temperature and subsequently the sound speed. According to acoustic theory [39], when a wave crosses an interface between two different media, some acoustic energy is reflected. In reacting flows, the density of the flow before and after the flame varies significantly. Therefore the acoustic wave passing through a flame resembles a wave crossing an interface between two different media. Figure 3 shows the piecewise continuous acoustic perturbation
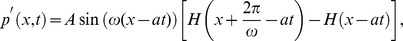
where *A* is again the maximum perturbation amplitude, 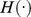 is the Heaviside function, and 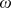 is the angular frequency (set to give 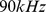 in this example).

**Figure 4:**
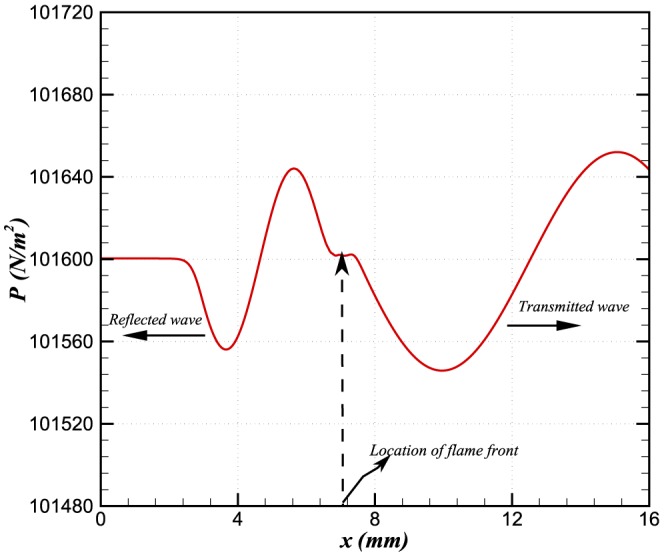
Transmitted and reflected waves.

**Figure 5:**
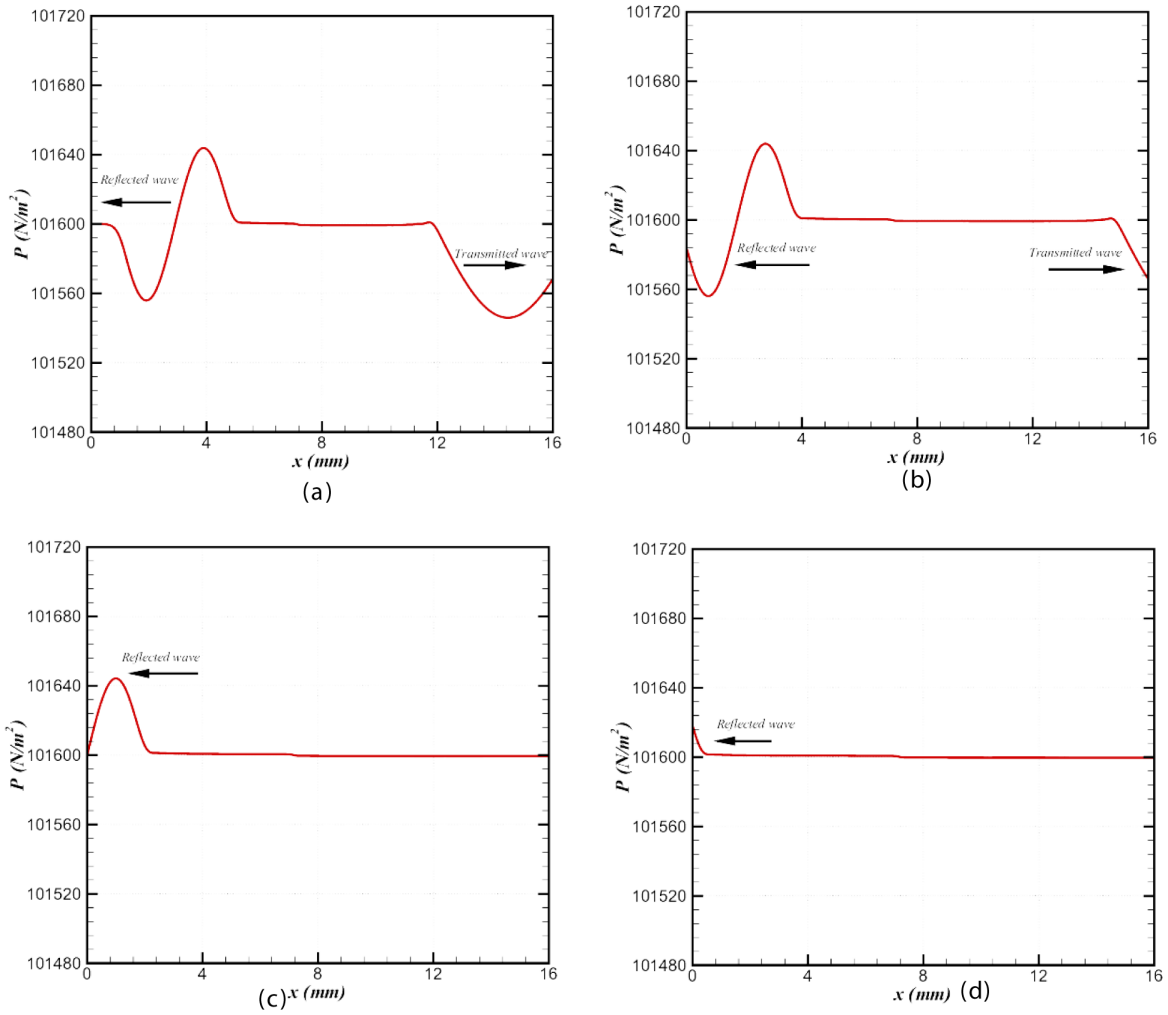
Snapshots of both waves at four different intervals: **(a).** 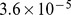 **sec, (b).** 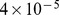 **sec, (c).** 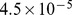 **sec, and (d).** 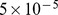 **sec.**

We observe that the acoustic wave is partially reflected when it hits the flame as shown in Figure 4. The reflection of the wave depends upon the product of density and sound speed in the media via *the acoustic impedance* [39]. The relation between the reflected and incident waves is established by the reflection coefficient, given by [39]:
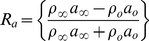
(7)where the 0 and 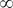 subscripts refer to the hot and cold sides of the flame, respectively. If the amplitudes of incident, transmitted and reflected waves are *I*, *T* and *R*, respectively, we can write
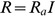
(8)

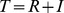


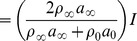
(9)The above relationships are derived for two media with different speeds of sound and density. The results of our simulation have shown that the amplitude of the reflected and transmitted waves are in agreement with analytical calculations obtained from Equations 8 and 9. The single wave simulation was performed for a simulation time of 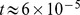 sec, corresponding to 1.304 acoustic transit times (based on the cold flow variables) to observe attenuation or amplification in the transmitted and reflected waves. Figures 5(*a*)–(*d*) show the waves at different time intervals, and we observe that both waves travel smoothly out of the domain without any further change to amplitude or frequency. The nonreflecting character of the inlet and outlet boundaries is evident in figures 5(a)–(d); Separate tests have demonstrated that the reflection coefficients for this boundary condition is 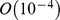 for physical waves, and 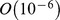 for numerical waves [35].

The amplitudes of the reflected wave and the transmitted wave are approximately 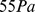 and 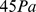, respectively, as shown in the figure 4. We define relative errors in the incident and reflected waves as
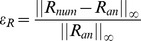


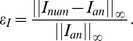
The subscripts 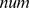 and 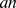 refer to the numerical and analytic result, respectively. We find that for our simulations, 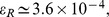 with a similar figure for 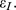 Rather than be a product of a non-linear phenomenon, this figure is more likely a result of the manner in which the amplitudes are measured—the wave peak almost never exactly collocates on a grid point, and so there is a small phase error induced in estimating the peak amplitude. Notwithstanding the foregoing argument, the error is small and the essential constancy of 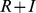 leads us to conclude that the acoustic wave has been neither amplified nor attenuated during its transit of the nonequilibrium region of the flow. This test has been repeated a number of times with different amplitudes and frequencies. The results were the same as those reported here.

### 0.5 Effect of a single wave on the rate chemistry

**Figure 6:**
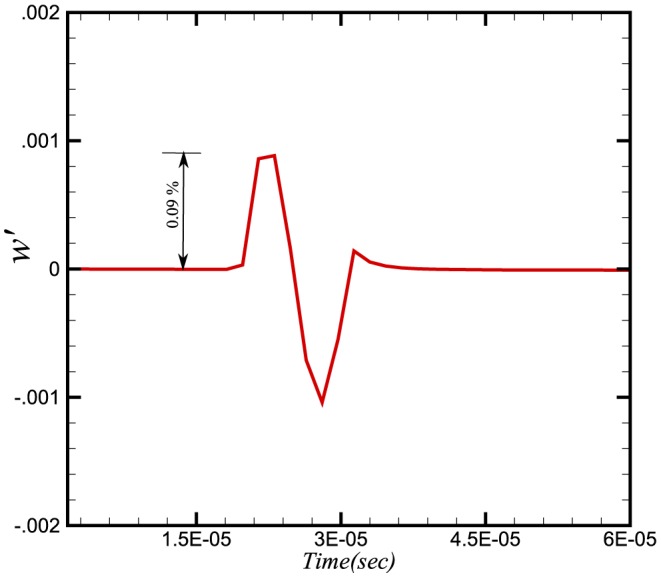
Relative change in reaction rate of 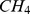.

**Figure 7:**
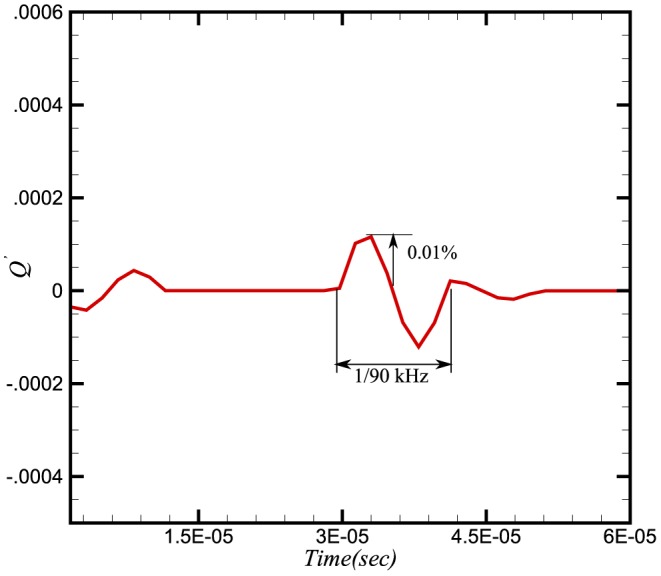
Relative change in heat release.

**Figure 8:**
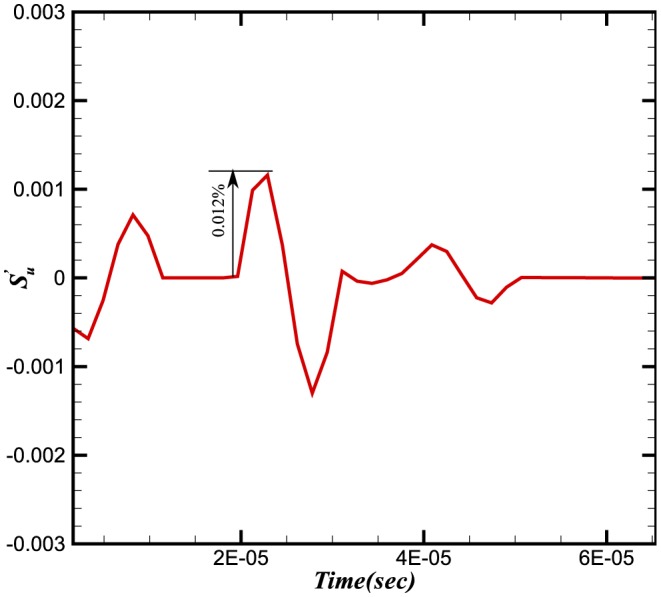
Relative change in burning velocities.

To study the effect of pressure waves on combustion chemistry, we have examined the response of the heat release, the reaction rate and the burning velocity to a number of imposed frequencies. Instantaneous integral values of reaction rate are obtained by integrating 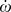 for a particular species over the domain length at each time step. Similarly the integral values of burning velocity and heat release are calculated. Figures 6, 7 and 8 show the time history of the relative change of the integral values of reaction rate of 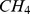, heat release and burning velocities respectively. The relative change is calculated using the following expressions:
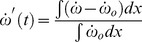
(10)

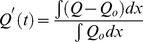
(11)

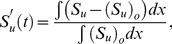
(12)where the suffix 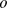 is used to refer to an acoustically quiescent benchmark solution i.e. no acoustic wave passing through the flame, and additionally 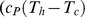 is a constant, 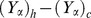 depends on which species you choose)
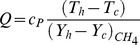


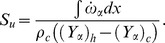

*c* and *h* refer to the cold and hot sides of the flame, respectively.

The relative changes in reaction rate, heat release and burning velocities are very small. A small perturbation in the integral values of heat release and burning velocities is also visible in figures 7 and 8 during initial stages (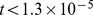 sec), which shows the effect on integral values when the wave is crossing the inlet boundaries. As the density and pressure are related through the equation of state, any fluctuation in pressure will also produce a fluctuation in the density. Consequently a fluctuation in the conservative form of species mass fraction 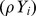 at the inlet will effect the integral values. This initial perturbation disappears once the wave has crossed the inlet (i.e. after 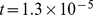 sec).

The perturbation in reaction rate and burning velocities are essentially instantaneous when the wave passes through the flame. However, a time delay can be seen in the heat release, which is due to the time scales related to the chemical reaction. Although the study of a single wave did not provide any direct effect of combustion on the amplification or attenuation of the acoustic wave, the perturbations in these three parameters may feed some energy to the subsequent acoustic waves.

### 0.6 Effect of multiple waves on rate chemistry

In this section, we extend our study to that of a high frequency wave train propagating through the flame structure. The purpose of this test is to identify additional effects arising from the coupling of the incoming waves to the flame, such as (say) standing waves local to the reaction zone. The simulation is run for a sufficient time 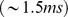 to ensure that at least 3 waves have crossed the flame thickness. Low frequency acoustic waves produce only negligibly small differential pressure gradients across the flame; such waves are felt by the flame essentially as a uniform background pressure oscillation. It is difficult to see how such a bulk effect could give rise to significant changes in the flame structure. Consequently, we restrict our attention to comparatively high frequencies: 3 kHz, 5 kHz, 8 kHz and 10 kHz are chosen. In order to study the sensitivity of the flame to both amplitude and frequency, each frequency is simulated for three different pressure perturbations of amplitudes 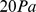, 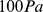 and 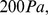 corresponding to sound pressure levels of 140 dB, 168 dB and 180 dB, respectively.

#### 0.6.1 Configuration 1. Frequency fixed and amplitude varied

**Figure 9:**
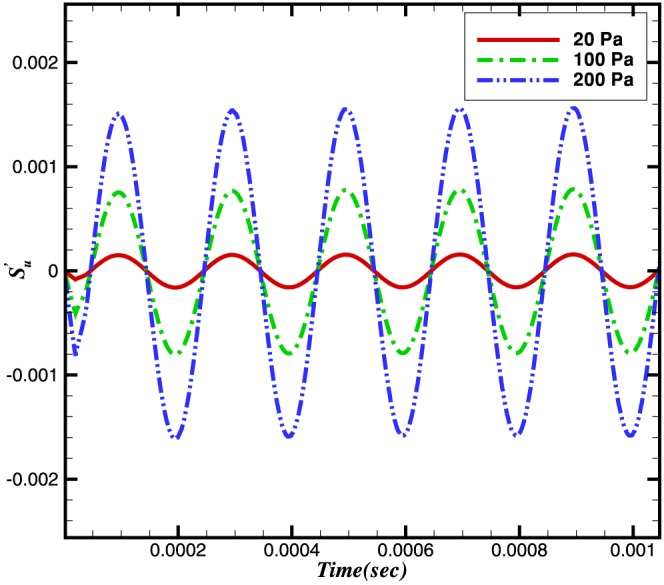
Relative change in the burning velocities at different amplitudes and a constant frequency of 5 kHz.

**Figure 10:**
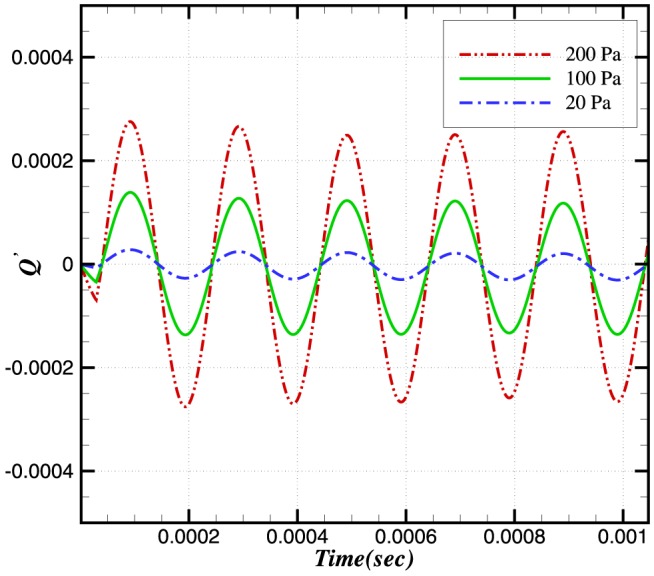
Relative change in the heat release at different amplitudes and a constant frequency of 5 kHz.

Figures 9 and 10 show the dependences on pressure of the burning velocity and heat release on the pressure.

**Figure 11:**
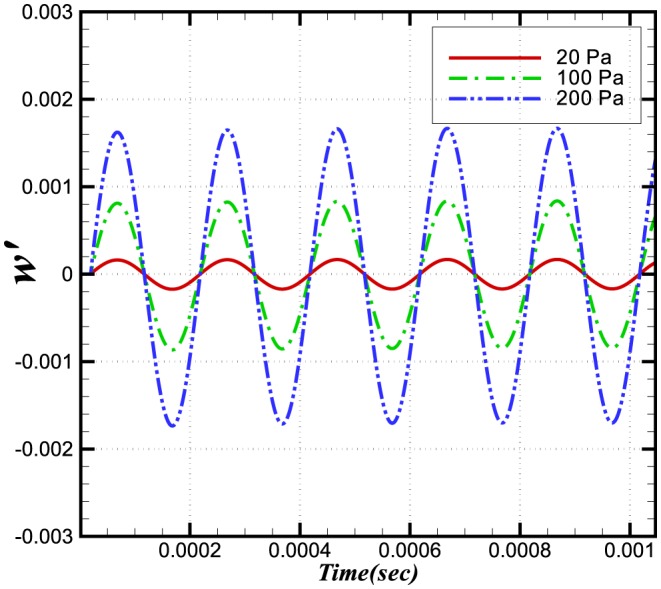
Relative change in the reaction rate of 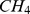 at different amplitudes and a constant frequency of 5 kHz.

**Figure 12:**
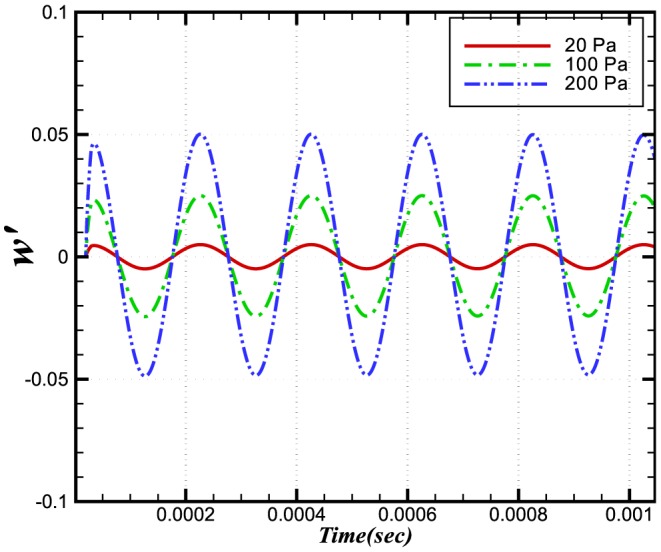
Relative change in the reaction rate of 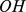 at different amplitudes and a constant frequency of 5 kHz.

**Figure 13:**
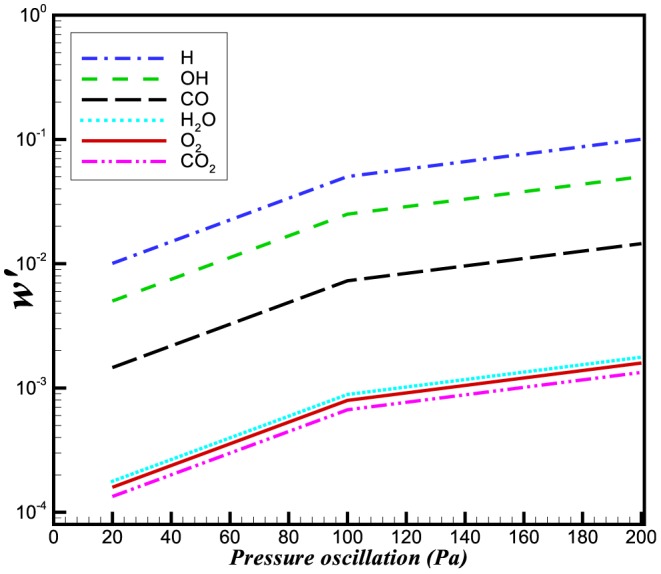
Relative change in the reaction rates of different species at a constant frequency of 5 kHz.

The reaction rate integrals of 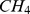 and 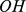 are shown in figures 11 and 12. It can be seen that the relative change in the reaction rate of 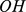 (and hence its integral) is larger than that associated with 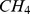. The relative change in the reaction rates of a number of other species is also shown in figure 13. Although the relative change in the 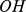 and 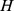 is moderate, the net effect of these species in terms of the heat release is very small.

#### 0.6.2 Configuration 2. Frequency varied and amplitude fixed

**Figure 14:**
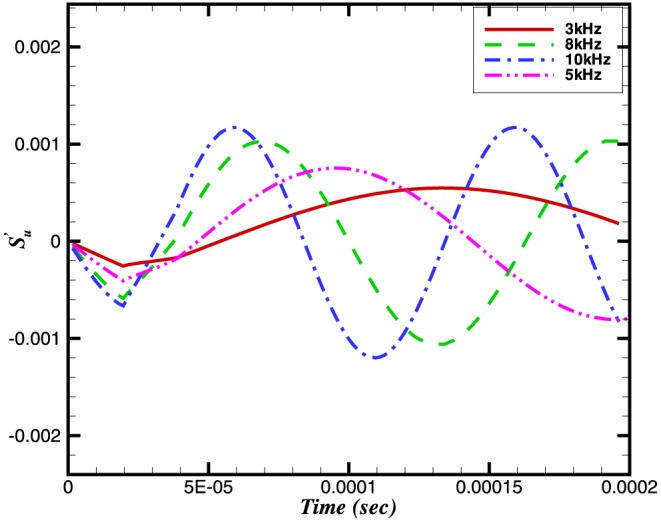
Relative change in the burning velocities at different frequencies and a constant amplitude of 100 Pa.

**Figure 15:**
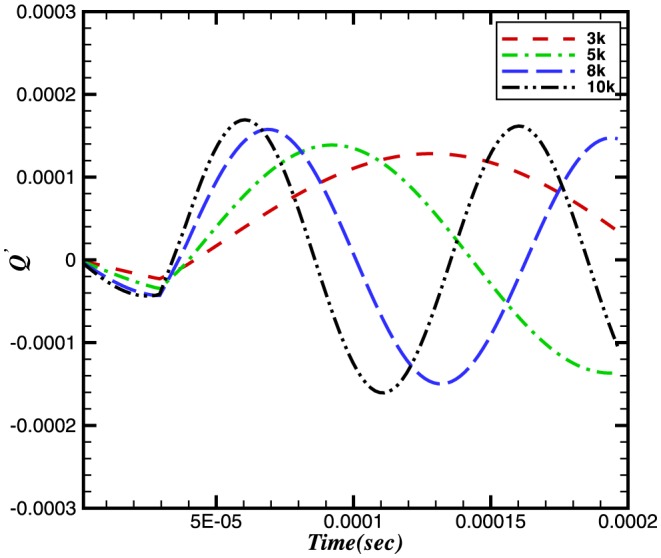
Relative change in the heat release at different frequencies and a constant amplitude of 100 Pa.

**Figure 16:**
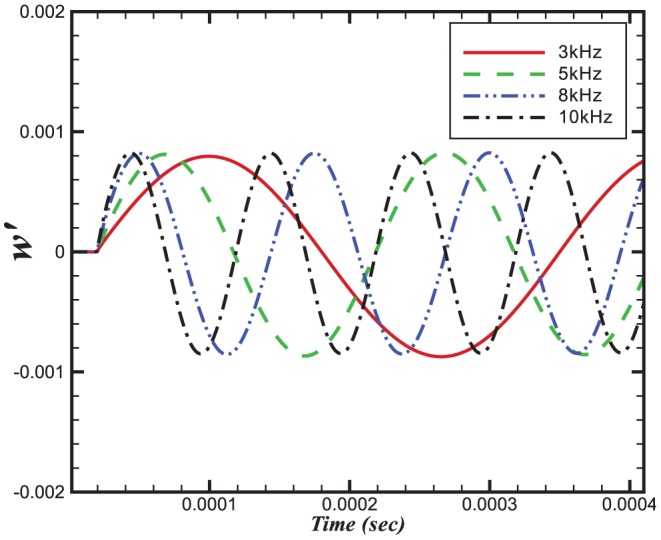
Relative change in the reaction rate of 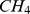 at different frequencies and a constant amplitude of 100 Pa.

**Figure 17:**
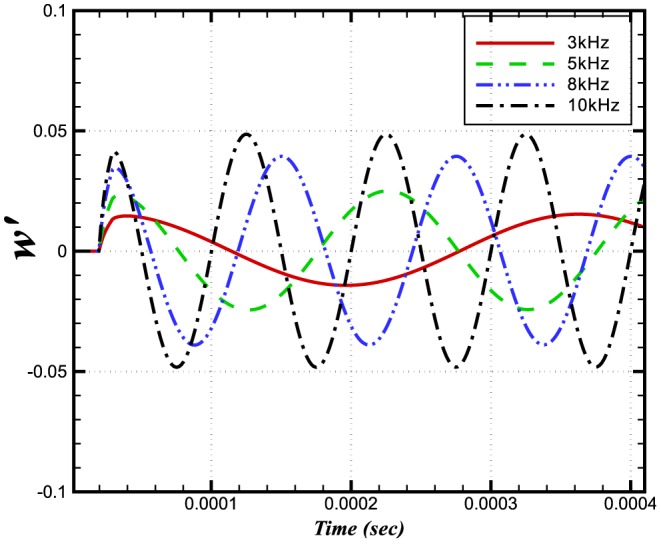
Relative change in the reaction rate of 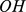 at different frequencies and a constant amplitude of 100 Pa.

**Figure 18:**
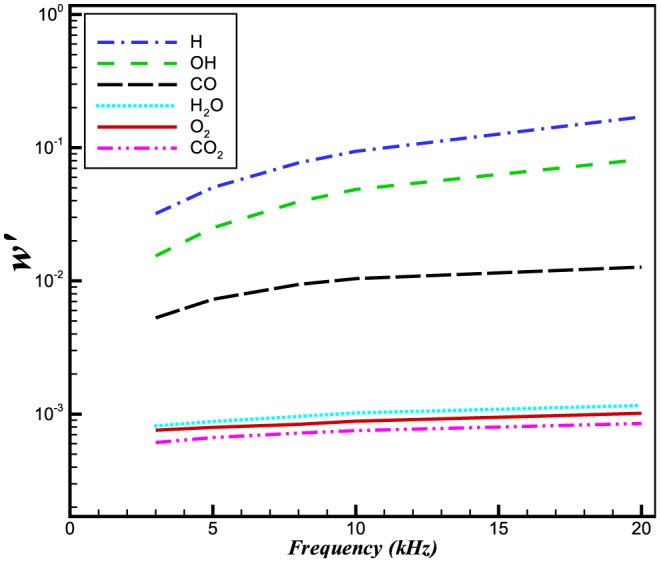
Relative change in the reaction rates of different species at a pressure perturbation of 100 Pa.

The relative changes in burning velocity and heat release for 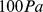 perturbations imposed at different frequencies are shown in figures 14 and 15, respectively. Interestingly, both quantities exhibit a frequency dependence, with their peak values increasing with increasing frequency. This effect appears to result from a change in the flame structure. Evidence for this observation comes from figures 16 and 17 which, between them show different sensitivities on the 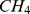 and 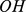 production rates. Additionally, figure 18 depicts the maximum change in production of a number of other species, with respect to the incident wave frequency. This figure shows that there exists no simple relation between the molecular weight of a species and its relative change. The 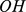 and 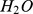 curves, for example share very similar molecular weights, but exhibit very different behaviours with respect to imposed frequency. We conclude from this that the change in flame speed cannot result simply from the pressure gradient acting preferentially on the light species.

#### 0.6.3 Higher frequency effects

In the foregoing parts of the paper, the flame thickness is small compared to the incident acoustic wavelength (i.e. a 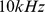 wave has a wavelength 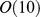 times greater than the simulated flame thickness of approximately 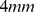). In such cases the effect of the pressure wave will produce very small pressure gradients across the flame. To obtain a more realistic measure of the pressure fluctuation on the flame, we have extended the range of high frequencies to ensure a more comparable relation between flame thickness and wavelength.

Following McIntosh [40], we define the ratios of time and lengthscale for flame-acoustic interaction as:
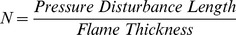
(13)

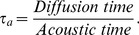
(14)Using the Mach number 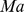 Based on the flame speed, both time and length scales can be related as [40]:
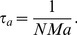
(15)For a harmonic wave, the disturbance length is taken as half of the wavelength: for 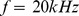 (say) the disturbance length is 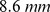 based upon the initial sound speed in the fuel/air mixture of 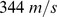. The parameter 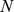 is critical in establishing the flame-acoustic interaction. Strong pressure effects on flame/acoustic configurations with small 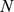 arise as a result of sharp pressure gradients across the flame [41]. McIntosh [42] has also observed that the effect of pressure gradients will be more important when 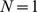 and 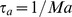.

**Figure 19:**
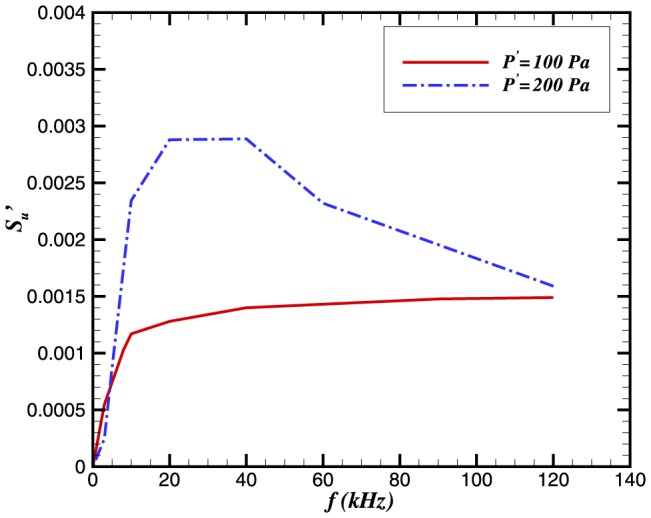
Relative change in the burning velocity vs frequency.

**Figure 20:**
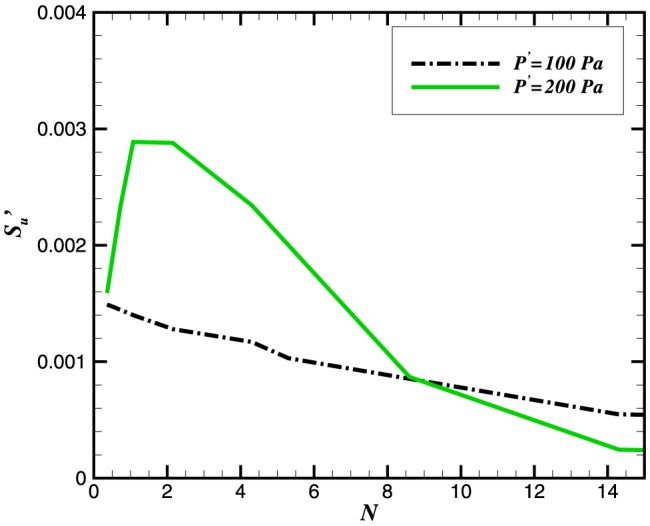
Relative change in the burning velocity vs length scale ratio *N*.

We have adopted an alternate form to define the acoustic time scale ratio 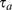 in terms of frequency:
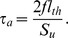
The above expression shows a direct relation to frequency of the incident wave. In our analysis of high frequencies, we have found that the effect of pressure perturbations increases when *N* is decreased. The relative change is a maximum when *N* reaches unity. Figure 19 and 20 depict the maximum values of 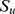 with pressure perturbations of 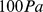 and 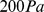. Pressure perturbations of 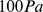 do not appear to have a significant effect on the flame speed perturbation. This is in marked contrast to the 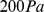 case, for which there exists a marked peak for 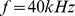 (corresponding approximately to 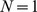). This lends further strength to the notion that acoustic influences are not restricted just to preferential acceleration of the light species; the pressure gradients seen by a flame are the same for a wave of amplitude *p* and frequency *f* as they are for a wave of amplitude 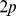 and frequency 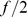
—yet the figures show no such correspondence in their profiles. Hence, it appears that the pointwise value of pressure (as well as its gradient) is important to the flame. This is ostensibly a surprising result, since a 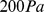 perturbation only corresponds to 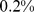 os the total pressure the flame sees. Nevertheless, this figure is approximately consistent with the flame speed changes observed. For oscillations of 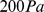 we see that a peak change is near 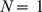, and a downward trend is observed for 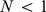. This shows that for a value of 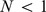, the effect of the pressure amplitude becomes less significant.

We have not studied further frequencies beyond 120 kHz because these frequencies are not often found (i.e. 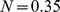) in practical applications. Although large fluctuations may result in extinction and re-ignition of the flame, the relative change in the burning velocities in our simulations is not substantial for the range of pressure fluctuations studied.

## Conclusions

A study of a one-dimensional flame with relatively detailed chemistry is carried out with oscillating pressure inflow conditions. The effects of a single wave and a continuous wave train on the reaction rate, heat release and burning velocities is studied. We have observed that these three parameters exhibit sensitivity both to the amplitude and frequency of the acoustic wave. Using pressure perturbations of 20 Pa, 100 Pa and 200 Pa, we have observed that fluctuations in heat release, reaction rate and burning velocities increase with an increase in pressure. The effect of frequency is better understood in terms of the ratio of acoustic wavelength and flame thickness. We have observed that when this ratio is near unity the acoustic effects are more significant. When this ratio is decreased i.e. 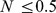 the change in burning velocity perturbation is very small. The relative changes in burning velocity and heat release are very small (less than 0.1%) in all cases. The effect of the acoustic waves on the reactions is not uniform however, as indicated by the relatively larger changes in minor species such as 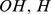 and 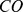.

The effect of a non-equilibrium background flow on acoustic wave propagation was examined. Unlike other studies(i.e. [12], [13], [16], [17], [18]) , we could find no evidence of wave attenuation/amplification resulting from the wave-flame interaction

For the detailed study of flame behaviour subjected to acoustic oscillations, a 1D study may not be enough and a better understanding can be developed from two or three-dimensional simulation. The effects of change in flame area (i.e. wrinkling) and subsequent burning rate are not visible in the 1D case. Additionally, we have carried out our simulation with an equivalence ratio 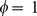; the flame response with different equivalence ratios will give a fuller understanding of the sensitivity of the flame to the acoustic perturbations.
